# When Trust and Privacy Pull in Opposite Directions: Consumer Decision Making with Conversational AI

**DOI:** 10.3390/bs16101854

**Published:** 2026-10-09

**Authors:** Fengying Zhuo, Junqing Zhu, Chenshu Liu

**Affiliations:** College of Fashion and Design, Donghua University, Shanghai 200051, China

**Keywords:** conversational AI, consumer decision making, algorithmic trust, privacy concern, AI literacy, purchase intention

## Abstract

Drawing on the Stimulus–Organism–Response (S-O-R) framework, this study examines how consumers’ perceptions of information quality, personalization, explainability, and responsiveness are associated with purchase intention through their relationships with perceived usefulness, algorithmic trust, and privacy concern, while considering AI literacy as a boundary condition. Data from 588 Chinese online shoppers who completed a standardized shopping task through direct interaction with a conversational AI shopping assistant were analyzed using structural equation modeling. The results show that all four consumer-perceived conversational-AI cues are positively associated with perceived usefulness and algorithmic trust, whereas perceived personalization is also positively associated with privacy concern. Perceived usefulness and algorithmic trust are positively associated with purchase intention, whereas privacy concern shows a negative conditional association with purchase intention in the structural model. Personalization shows competing indirect associations with purchase intention: positive indirect associations through usefulness and trust coexist with a negative indirect association through privacy concern. The positive trust–intention association and the negative conditional privacy concern–intention association are stronger at higher levels of AI literacy, whereas the usefulness–intention interaction is statistically compatible with practical equivalence relative to the specified study-specific bound. These findings indicate that consumer responses to conversational AI reflect competing benefit–risk evaluations rather than a uniformly positive acceptance process, and that AI literacy is differentially associated with the strength of the trust–intention and privacy concern–intention relationships.

## 1. Introduction

Artificial intelligence (AI) is increasingly involved in consumer decision making, transforming online shopping from a largely one-way recommendation process into an interactive form of decision support (Balakrishnan & Dwivedi, 2024; Sidlauskiene et al., 2023). Conversational AI shopping assistants allow consumers to articulate preferences in natural language, refine decision criteria, compare alternatives, and request explanations as their needs evolve (P.-F. Hsu et al., 2023). This shift changes the role of AI in the consumer journey. Rather than functioning merely as an invisible recommendation mechanism, conversational AI becomes an interactive decision agent whose information, recommendations, and responses are directly evaluated by consumers. Consequently, consumer decisions depend not only on what products are recommended but also on how consumers psychologically interpret their interactions with AI.

These interpretations are not necessarily uniformly positive or negative. Consumers may perceive AI-generated information as accurate and useful, regard personalized recommendations as relevant to their needs, and develop trust in the system’s ability to support their decisions (Ashfaq et al., 2020; C.-L. Hsu & Lin, 2023; Kasilingam, 2020; Shin, 2021). At the same time, the same interaction can make data collection and inference more salient, particularly when recommendations appear highly personalized (Aguirre et al., 2015). Consumers may therefore appreciate the benefits of AI assistance while remaining concerned about how their personal information is collected, inferred, or used. Conversational AI thus creates a decision environment in which benefit-oriented and risk-oriented evaluations can coexist (X. Chen et al., 2022). Understanding consumer responses to AI consequently requires moving beyond a simple acceptance-versus-rejection perspective and examining how competing psychological evaluations are associated with purchase intention.

Despite growing research on chatbots and AI-enabled commerce (X. Luo et al., 2019; Shahzad et al., 2024), this coexistence of positive and negative evaluations remains insufficiently integrated into explanations of consumer responses. Benefit-oriented mechanisms such as usefulness and trust and risk-oriented mechanisms such as privacy concern have often been examined in separate research streams. This separation is particularly consequential for personalization. Tailored recommendations can increase relevance and perceived value, while trust plays an important role in consumers’ responses to personalized communication (Bleier & Eisenbeiss, 2015; Kalaignanam et al., 2018). Yet personalization may simultaneously signal that an AI system possesses, collects, or infers substantial information about the consumer, thereby heightening privacy concern (X. Chen et al., 2022). The association between personalization and purchase intention may therefore reflect competing psychological pathways rather than a uniformly beneficial pattern. Examining these pathways simultaneously is important for explaining why favorable evaluations of an AI feature may not necessarily correspond to equally strong purchase intention.

A second issue concerns the alignment between psychological judgments and purchase intention. Favorable and unfavorable evaluations of AI may differ in how strongly they are associated with purchase intention. Prior research further shows that stated concerns and intentions do not always translate consistently into subsequent behavior (Norberg et al., 2007), underscoring the importance of distinguishing intention-level associations from actual behavior. This raises an important question: under what conditions are consumers’ evaluations of AI more strongly aligned with their purchase intentions? AI literacy may provide one such boundary condition. It encompasses the knowledge and competencies required to understand, use, evaluate, and critically engage with AI systems (Ng et al., 2021; Pinski & Benlian, 2024). Rather than simply making consumers more favorable toward AI, greater AI literacy may help them interpret the implications of their existing judgments and connect those judgments more consistently to purchase intention. Importantly, this alignment may differ across psychological evaluations. Trust and privacy concern involve judgments about uncertain system capabilities and data-related consequences, whereas perceived usefulness already contains a relatively direct assessment of the instrumental value of AI-assisted decision making. AI literacy may therefore strengthen some evaluation-to-intention relationships without uniformly amplifying all evaluations of AI.

A third issue concerns how characteristics of conversational AI generate these different psychological responses. Consumers interact with conversational AI through a combination of informational and interactional cues rather than through isolated technological features (Shahzad et al., 2024). They evaluate whether the information provided is accurate and relevant, whether recommendations correspond to their individual needs, whether recommendation logic is understandable, and whether the system responds appropriately as the interaction develops (Ashfaq et al., 2020; C.-L. Hsu & Lin, 2023). These characteristics may not operate through identical psychological processes. Some can increase the instrumental value of AI-assisted decision making, some can provide cues that support trust, and some—particularly personalization—can simultaneously make privacy risks more salient. An integrated framework is therefore needed to explain how multiple characteristics of conversational AI give rise to parallel and potentially competing psychological evaluations.

To address these issues, this study develops an extended Stimulus–Organism–Response (S-O-R) framework of consumer decision making with conversational AI. The S-O-R perspective explains how external technological or environmental stimuli shape internal psychological states that subsequently influence behavioral responses and has been widely applied in online retail contexts (Eroglu et al., 2001) and more recently to AI-chatbot quality and consumer responses (Elayat & Elalfy, 2025). In the present framework, information quality, perceived personalization, perceived explainability, and perceived responsiveness represent consumer-perceived conversational-AI cues on the stimulus side of the framework; perceived usefulness, algorithmic trust, and privacy concern represent parallel organismic states; and purchase intention represents the intention-level response construct. Their stimulus-side positioning is conceptual and does not imply that these characteristics were experimentally manipulated or objectively varied across participants. Perceived usefulness and algorithmic trust capture benefit-oriented evaluations, whereas privacy concern captures a concurrent risk-oriented evaluation. AI literacy is further conceptualized as a boundary condition affecting the extent to which these psychological judgments are translated into purchase intention.

This study makes three contributions to understanding purchase-intention formation in AI-mediated decision environments. First, it conceptualizes consumer responses to conversational AI as a process involving concurrent benefit and risk evaluations rather than a uniformly positive technology-acceptance process. By modeling perceived usefulness, algorithmic trust, and privacy concern as parallel psychological states, the study captures the possibility that consumers can simultaneously recognize the value of AI assistance and experience concerns about its consequences. Second, it highlights the competing benefit- and privacy-related associations surrounding personalization. Perceived personalization may be positively associated with usefulness and trust while also being positively associated with privacy concern, creating opposing psychological pathways to purchase intention. Third, the study conceptualizes AI literacy as a boundary condition for evaluation-to-intention alignment rather than as a universal amplifier of AI acceptance. By examining whether AI literacy differentially moderates the associations of usefulness, trust, and privacy concern with purchase intention, the study provides a more differentiated account of when psychological evaluations of AI are more strongly associated with purchase intention.

## 2. Literature Review

### 2.1. Conversational AI as a Consumer Decision Environment

Rather than treating conversational AI characteristics as an exhaustive set of objective technological features, this study focuses on four consumer-perceived cues that are particularly proximal to AI-supported shopping decisions. Conversational AI shopping assistants are AI-enabled systems that interact with consumers through natural language and support product search, comparison, evaluation, and choice (Mariani et al., 2023). Unlike conventional recommendation systems, they allow consumers to refine preferences, request explanations, and adjust decision criteria through continuous interaction. As a result, consumers do not merely receive recommendations; they actively interpret and respond to information, personalized suggestions, explanations, and conversational feedback throughout the decision process. Conversational AI can therefore be viewed as an interactive decision environment in which consumer-perceived informational and interactional cues are associated with consumers’ psychological evaluations. The selection of the four focal cues follows three considerations. First, each can be directly perceived and evaluated by consumers during their interaction with a conversational AI shopping assistant. Second, together they capture both the substantive content of decision support and the interaction process through which that support is understood and adapted. Third, they are theoretically relevant to the psychological evaluations examined in this study, including instrumental value, system reliability, and privacy-related concern.

Four characteristics are particularly relevant to this process. Information quality refers to the accuracy, relevance, completeness, and consistency of the information provided by the AI. Because consumers rely on such information when comparing alternatives and evaluating recommendations, information quality provides an important basis for judging the value and reliability of AI-supported decisions. Perceived personalization captures the extent to which recommendations are adapted to individual needs, preferences, and changing decision criteria. Research on conversational agents shows that personalized product recommendations can incorporate consumers’ individual product preferences (Rhee & Choi, 2020), making personalization an important cue of how closely the system responds to the individual consumer.

Perceived explainability refers to the extent to which consumers can understand why particular products are recommended and how those recommendations correspond to their needs. From a user-centered perspective, explainability is associated with the understandability, completeness, and transparency of AI-generated explanations (Haque et al., 2023). It therefore enables consumers to evaluate not only the recommendation outcome but also the reasoning underlying it. Perceived responsiveness, in turn, reflects whether the AI responds promptly, appropriately, and coherently as the interaction develops. Response timing and the ability to sustain an appropriate conversational exchange are important dimensions of human–chatbot interaction (Gnewuch et al., 2022).

Together, these characteristics represent distinct but complementary consumer-perceived conversational-AI cues through which consumers interpret conversational AI. Information quality and personalization primarily concern what decision support consumers receive, whereas explainability and responsiveness concern how that support is communicated and adapted during interaction. These four cues are not intended to constitute an exhaustive taxonomy of conversational AI characteristics; rather, they represent a theoretically focused set of task-relevant decision-support cues spanning both the content and interaction process of AI-assisted shopping. Their relevance lies in their associations with consumers’ evaluations of instrumental value, system reliability, and potential privacy-related risks.

### 2.2. The S-O-R Framework in AI-Mediated Consumer Decision Making

The S-O-R perspective conceptualizes relationships among external technological or environmental stimuli, internal psychological states, and behavioral responses. In the present cross-sectional study, this framework is used to organize the hypothesized associations among the focal constructs rather than to establish their causal or temporal ordering (Eroglu et al., 2003). This perspective is well suited to conversational AI shopping because consumers do not respond directly to technological features alone. Instead, they interpret informational and interactional cues, form psychological evaluations of the AI-assisted decision experience, and translate these evaluations into subsequent behavioral intentions (Cheng et al., 2021).

Accordingly, information quality, personalization, explainability, and responsiveness are conceptualized as consumer-perceived conversational-AI cues occupying the stimulus side of the S-O-R framework, while perceived usefulness, algorithmic trust, and privacy concern represent organismic states and purchase intention represents the intention-level response construct (Vafaei-Zadeh et al., 2025). This stimulus-side classification refers to their conceptual role in the S-O-R framework rather than to experimentally manipulated external stimuli. Rather than treating these psychological states as sequential or mutually exclusive, this study models them as parallel evaluations.

### 2.3. Coexisting Benefit- and Risk-Oriented Psychological Evaluations

Perceived usefulness reflects consumers’ belief that conversational AI improves shopping efficiency, product comparison, and decision quality (Davis, 1989). It represents an instrumental evaluation of whether AI assistance contributes value to the shopping task and has been shown to play an important role in translating consumers’ evaluations of chatbot features into subsequent behavioral responses (Zhu et al., 2023). Algorithmic trust, by contrast, refers to consumers’ willingness to rely on an AI system based on perceptions of its competence, reliability, consistency, and appropriate functioning (Glikson & Woolley, 2020). Because consumers cannot fully observe how AI recommendations are generated, such trust is often formed from observable system characteristics and users’ interpretations of AI performance (Gillath et al., 2021).

Perceived usefulness and algorithmic trust therefore represent two related but distinct benefit-oriented evaluations. Usefulness concerns the instrumental value that consumers expect to obtain from AI-supported decision making (Davis, 1989), whereas trust concerns whether consumers are willing to rely on the AI despite uncertainty about its underlying processes and outputs (Glikson & Woolley, 2020). A system may thus be perceived as useful without being fully trusted, while trust may also depend on judgments that extend beyond instrumental performance.

Importantly, treating perceived usefulness and algorithmic trust as parallel organismic evaluations does not imply that the two constructs are unrelated or that a sequential relationship between them is theoretically impossible. Rather, the parallel specification reflects their distinct evaluative objects within the present decision context. Perceived usefulness is primarily a task-oriented instrumental judgment concerning whether conversational AI improves shopping efficiency and decision quality, whereas algorithmic trust is a reliance-oriented judgment concerning whether the AI can be depended upon despite uncertainty about its underlying processes and outputs. Recent AI research also suggests that the structural relationship between usefulness and trust is not represented uniformly across contexts. Some studies model perceived usefulness as an antecedent of trust in generative-AI adoption (T. Luo & Cao, 2026), whereas research on AI-powered retail platforms has reported trust as an antecedent of perceived usefulness while treating both constructs as relevant to purchase intention (ElSayad & Mamdouh, 2024). These alternative specifications suggest that usefulness and trust are conceptually related but that a single directional ordering should not be assumed to be universal across AI-use contexts. Consumers may therefore form both evaluations in response to the same AI-supported interaction without one necessarily serving as a prerequisite for the other in the focal model.

Privacy concern represents a parallel risk-oriented evaluation. AI-enabled personalization often depends on information that consumers explicitly provide, that is observed through their behavior, or that is inferred during interaction. As a result, consumers may become concerned about how personal information is collected, managed, inferred, and subsequently used (Milne & Boza, 1999). Greater personalization can improve recommendation relevance while simultaneously making data collection and algorithmic inference more salient (Teepapal, 2025). The same feature may therefore generate both decision benefits and perceived privacy costs.

Consumers can consequently regard a conversational AI assistant as useful and trustworthy while still expressing concern about its use of personal information. Research on AI-driven personalization similarly suggests that usefulness, trust, and privacy concern can coexist rather than forming opposite ends of a single evaluative continuum (Teepapal, 2025). Accordingly, this study treats privacy concern as an independent risk-oriented psychological state that operates alongside benefit-oriented evaluations (Milne & Boza, 1999). Taken together, perceived usefulness, algorithmic trust, and privacy concern capture three conceptually distinct evaluations that consumers may form during the same AI-supported shopping interaction: whether the AI provides instrumental value for the shopping task, whether its recommendations can be relied upon, and what privacy implications are associated with the underlying use of personal information. Modeling these states in parallel does not assume that they are statistically independent or preclude possible relationships among them; rather, it avoids imposing a necessary temporal ordering among distinct psychological evaluations in the focal cross-sectional model.

### 2.4. Content-Related and Interaction-Process Cues

The four conversational AI characteristics can be distinguished according to whether they primarily concern the substance of decision support or the process through which that support is delivered. Information quality and personalization are primarily content-related cues because they determine the accuracy, relevance, and individual fit of the information and recommendations consumers receive. Prior research on recommendation agents shows that recommendation content and personalization can shape consumers’ product evaluations, trust, and subsequent buying responses (Ampadu et al., 2022; Chang & Park, 2024; Xiao & Benbasat, 2007).

Explainability and responsiveness, by contrast, are primarily interaction-process cues. Explainability helps consumers understand recommendation logic and evaluate decision trade-offs, while responsiveness reflects how effectively the AI responds and adapts during interaction. Explanation-related features can improve product diagnosticity and perceived decision quality (Xu et al., 2014), whereas responsiveness can enhance usefulness and other evaluations that subsequently influence trust and behavioral responses (Mohamed Abd-El-Salam & Hassan Youssef, 2026). This content–process distinction is used here as an organizing framework for the four selected consumer-perceived cues rather than as an exhaustive taxonomy of conversational-AI characteristics.

This distinction suggests that AI characteristics may be associated with purchase intention through different routes. Content-related cues may retain associations with purchase intention beyond consumers’ general evaluations of the AI because they directly shape the information and products being considered (Ampadu et al., 2022). Interaction-process cues, in contrast, are more likely to be associated with purchase intention through psychological evaluations of the AI-supported decision process, particularly perceived usefulness and trust (Mohamed Abd-El-Salam & Hassan Youssef, 2026; Xu et al., 2014).

### 2.5. AI Literacy and Evaluation-to-Intention Alignment 

AI literacy refers to the knowledge, skills, and critical capabilities that enable individuals to understand AI systems, use them effectively, evaluate their outputs, and recognize their broader implications (Carolus et al., 2023; Laupichler et al., 2023). In conversational shopping, these competencies help consumers formulate requests, assess recommendations, identify possible limitations, and make more informed judgments about AI-generated outputs. Research further suggests that AI literacy can influence how users interact with generative AI and the quality of the outcomes they obtain (Knoth et al., 2024).

In the present study, AI literacy is conceptualized as an overarching self-perceived capability rather than as a set of separately modeled competencies. Recent research conceptualizes AI literacy as encompassing complementary competencies related to understanding AI, interacting effectively with AI systems, critically evaluating AI-generated content, and recognizing ethical and societal implications (Lintner, 2024). Within the conversational shopping context, understanding AI, formulating effective requests, evaluating generated information, detecting potential errors, and recognizing broader data-related and algorithmic issues are therefore treated as related aspects of consumers’ perceived capacity to engage with AI critically and effectively. Although these competencies are distinguishable, their shared conceptual focus provides a rationale for examining an overarching AI literacy construct. Accordingly, the present study adopts a single-factor operationalization, consistent with its reported measurement results, while recognizing that alternative multidimensional structures may capture additional distinctions among these competencies (Koch et al., 2024).

Importantly, greater AI literacy does not necessarily lead to uniformly stronger acceptance of AI. AI knowledge and perceived understanding can shape trust formation, while greater literacy may also encourage more selective and critical reliance on AI systems (Pan et al., 2025). This suggests that AI literacy may matter not only for how consumers evaluate AI, but also for how strongly established evaluations are associated with subsequent purchase intentions.

This evaluation-to-intention alignment is particularly relevant for trust and privacy concern. Algorithmic trust involves uncertainty about whether an AI system is sufficiently competent and reliable to warrant reliance, whereas privacy concern involves judgments about data collection, inference, and possible secondary use. Greater AI literacy may help consumers interpret these judgments more clearly and connect them more consistently to purchase intentions (Ma & Chen, 2023; Pan et al., 2025). By contrast, perceived usefulness already reflects a relatively direct instrumental assessment of whether AI improves search, comparison, or decision quality, leaving less room for additional literacy to alter the strength of the usefulness–purchase intention association.

Emerging evidence also indicates that the effects of AI literacy can differ across evaluative pathways rather than operating uniformly (Kim et al., 2026). Accordingly, this study conceptualizes AI literacy as a boundary condition for evaluation-to-intention alignment. Its moderating role is expected to depend on the nature of the psychological evaluation involved rather than to operate as a general amplifier of AI acceptance.

## 3. Research Hypotheses

### 3.1. Conversational AI Characteristics and Perceived Usefulness

Conversational AI characteristics are expected to be associated with perceived usefulness through their relevance to the efficiency and quality of consumers’ shopping decisions. High-quality information reduces uncertainty and the need for additional verification. When AI-generated information is accurate, relevant, and reliable, consumers can evaluate alternatives more efficiently and make better-informed choices. Complete and consistent information may further reduce the resources consumers need to devote to checking or correcting AI-generated product information. Prior research on generative AI chatbots similarly shows that information quality is positively associated with perceived usefulness (Niu & Mvondo, 2024).

Personalization is expected to be positively associated with usefulness by adapting recommendations to consumers’ preferences, needs, and situational criteria. By filtering irrelevant alternatives and increasing the fit between recommendations and individual requirements, personalized decision support can reduce search effort and improve decision efficiency. It can also direct consumers’ attention toward alternatives that more closely correspond to their current decision criteria, thereby increasing the instrumental relevance of AI-supported decision assistance. Research on e-commerce recommendation systems further indicates that perceived usefulness is closely related to how well personalization matches consumer characteristics and the complexity of the shopping task (Nguyen & Hsu, 2022). Thus, when conversational AI adjusts its recommendations to consumers’ changing criteria, it is more likely to be perceived as useful.

Explainability contributes to usefulness by making the rationale underlying AI recommendations more accessible to consumers. When consumers understand why a product is recommended and how the recommendation corresponds to relevant product attributes or personal requirements, they can evaluate alternatives more efficiently and determine whether the recommendation is appropriate for their decision context. Clear explanations may therefore reduce the cognitive effort required to interpret AI-generated advice and provide additional diagnostic information for evaluating alternatives. Experimental research on explainable AI indicates that greater perceived explainability is associated with higher perceived usefulness (Hamm et al., 2023). Complementary research on generative AI also suggests that AI-supported interactions can reduce cognitive demands in complex tasks (Rehman et al., 2026). Accordingly, greater perceived explainability is expected to be associated with greater perceived usefulness.

Responsiveness contributes to usefulness by allowing consumers to refine requirements, resolve uncertainties, and obtain updated recommendations throughout the interaction. When conversational AI responds promptly and appropriately to changing needs, consumers can navigate the decision process more efficiently. Responsive interaction can also reduce interaction friction by allowing consumers to iteratively clarify their criteria rather than reformulating the shopping task from the beginning. Evidence from AI chatbot services similarly identifies responsiveness as an important antecedent of perceived usefulness (Le & Nguyen, 2025).

Together, these characteristics are expected to be positively associated with perceived usefulness through complementary informational and interactional routes. Accordingly:

**H1a.**  *Information quality is positively associated with perceived usefulness.*

**H1b.**  *Perceived personalization is positively associated with perceived usefulness.*

**H1c.**  *Perceived explainability is positively associated with perceived usefulness.*

**H1d.**  *Perceived responsiveness is positively associated with perceived usefulness.*

### 3.2. Conversational AI Characteristics and Algorithmic Trust

Consumers cannot fully observe how conversational AI generates recommendations and therefore infer its competence and reliability from observable characteristics of the interaction. The four consumer-perceived conversational-AI cues provide complementary signals for evaluating whether the AI can be relied upon in the shopping task. High-quality information provides performance-based evidence of system competence because accurate, relevant, and consistent information indicates that the system can perform its decision-support function reliably (Cardenas-Pauckar et al., 2026). Personalization provides evidence of adaptive fit: recommendations that reflect consumers’ preferences and changing criteria indicate that the system can recognize and adapt to individual needs (Komiak & Benbasat, 2006). Explainability reduces algorithmic opacity by making recommendation logic more understandable and transparent (Nilashi et al., 2016), thereby making the basis of recommendations more assessable to consumers. Responsiveness signals that the system can react appropriately to consumers’ requests and changing needs (J. Li et al., 2023) and therefore provides interaction-based evidence of operational reliability. Together, these characteristics provide distinct but complementary cues of competence, adaptive fit, assessability, and operational reliability that are expected to be positively associated with algorithmic trust. Accordingly:

**H2a.**  *Information quality is positively associated with algorithmic trust.*

**H2b.**  *Perceived personalization is positively associated with algorithmic trust.*

**H2c.**  *Perceived explainability is positively associated with algorithmic trust.*

**H2d.**  *Perceived responsiveness is positively associated with algorithmic trust.*

Personalization, however, can simultaneously be associated with privacy-related concerns. Highly individualized recommendations may make consumers more aware that the AI collects, accesses, or infers personal information. Because personalization relies on consumer data and preferences, greater personalization may increase the salience of data collection and raise concerns about how such information is obtained and used (Chellappa & Sin, 2005). Personalization is particularly relevant to privacy concern because individualized recommendations depend more directly on the collection, use, or inference of consumer-specific information. As recommendations become more closely tailored to personal preferences and changing criteria, the underlying use of personal data may also become more salient to consumers. This creates a benefit–risk tension in which consumers value tailored recommendations while also seeking control over their personal information (Canhoto et al., 2024). Personalization can therefore function as both a benefit-enhancing feature and a privacy-related cue. Accordingly:

**H3.**  *Perceived personalization is positively associated with privacy concern.*

### 3.3. Psychological States and Purchase Intention

Perceived usefulness, algorithmic trust, and privacy concern represent three distinct psychological evaluations that are expected to be associated with purchase intention. When consumers perceive AI-assisted shopping as useful, they are more likely to recognize its value in improving search and decision efficiency, which is expected to be positively associated with purchase intention (Pavlou, 2003). Algorithmic trust reflects consumers’ willingness to rely on the competence and reliability of AI-generated recommendations under uncertainty and is likewise expected to show a positive association with purchase intention (Song & Shin, 2024). Privacy concern operates in the opposite direction: concerns about the collection, inference, and use of personal information represent risk-oriented evaluations and are hypothesized to show a negative conditional association with purchase intention within the proposed model (Xie et al., 2020). These parallel evaluations capture potentially competing benefit- and risk-oriented associations with purchase intention. Accordingly:

**H4a.**  *Perceived usefulness is positively associated with purchase intention.*

**H4b.**  *Algorithmic trust is positively associated with purchase intention.*

**H4c.**  *Privacy concern shows a negative conditional association with purchase intention within the proposed structural model.*

### 3.4. Direct and Indirect Associations of Conversational AI Characteristics

The content–process distinction suggests that conversational AI characteristics may differ in the extent to which their associations with purchase intention operate through the proposed psychological states. Information quality and personalization concern the substantive information and recommendations consumers use to evaluate alternatives. Recommendation systems may be associated with purchase decisions through the alternatives consumers consider (X. Li et al., 2022), while personalization is associated with the relevance of tailored content (An & Ngo, 2025). Their associations with purchase intention may therefore remain evident after accounting for consumers’ general evaluations of the AI. Explainability and responsiveness, by contrast, primarily characterize how consumers understand and navigate the AI-supported interaction and may be more closely associated with purchase intention through psychological evaluations of the decision process (C. Chen et al., 2024; Le & Nguyen, 2025). Accordingly, the proposed distinction concerns the pattern of direct and indirect associations rather than whether any residual direct association is exactly zero. H5a and H5b address whether information quality and personalization retain direct associations with purchase intention after accounting for the proposed psychological pathways, whereas H5c and H5d address whether the associations of explainability and responsiveness with purchase intention are primarily reflected in the indirect component represented by the proposed organismic states. The specific indirect associations through perceived usefulness and algorithmic trust are specified separately in H6 and H7. Accordingly:

**H5a.**  *Information quality shows a positive direct association with purchase intention after accounting for the proposed psychological pathways.*

**H5b.**  *Perceived personalization shows a positive direct association with purchase intention after accounting for the proposed psychological pathways.*

**H5c.**  *Perceived explainability shows a positive total indirect association with purchase intention through the proposed organismic states.*

**H5d.**  *Perceived responsiveness shows a positive total indirect association with purchase intention through the proposed organismic states.*

Perceived usefulness represents a task-oriented, benefit-related evaluation that may account for one specific indirect association between conversational AI characteristics and purchase intention within the proposed model. Information quality is associated with the informational basis for choice, personalization with recommendation relevance, explainability with the evaluability of AI-generated recommendations, and responsiveness with interaction efficiency. Prior research similarly indicates that personalization, explainability, and responsiveness are associated with perceived usefulness (Hamm et al., 2023; Le & Nguyen, 2025; Nguyen & Hsu, 2022). H6 therefore specifies the perceived-usefulness component of the broader indirect-association pattern described above. Accordingly:

**H6a.**  *Information quality is expected to show a positive indirect association with purchase intention specifically through perceived usefulness.*

**H6b.**  *Perceived personalization is expected to show a positive indirect association with purchase intention specifically through perceived usefulness.*

**H6c.**  *Perceived explainability is expected to show a positive indirect association with purchase intention specifically through perceived usefulness.*

**H6d.**  *Perceived responsiveness is expected to show a positive indirect association with purchase intention specifically through perceived usefulness.*

Algorithmic trust represents a second benefit-oriented evaluation that may account for another specific indirect association within the proposed model. The characteristics of conversational AI may provide consumers with cues about system competence, relevance, transparency, and adaptive capability. Explainable recommendations may be positively associated with trust by making AI outputs easier to interpret (C. Chen et al., 2024), while responsiveness provides an additional signal of reliability during interaction (J. Li et al., 2023). Algorithmic trust, in turn, is expected to be positively associated with consumers’ willingness to rely on AI-generated recommendations when forming purchase intentions. H7 therefore specifies the algorithmic-trust component of the broader indirect-association pattern described above. Accordingly:

**H7a.**  *Information quality is expected to show a positive indirect association with purchase intention specifically through algorithmic trust.*

**H7b.**  *Perceived personalization is expected to show a positive indirect association with purchase intention specifically through algorithmic trust.*

**H7c.**  *Perceived explainability is expected to show a positive indirect association with purchase intention through algorithmic trust.*

**H7d.**  *Perceived responsiveness is expected to show a positive indirect association with purchase intention specifically through algorithmic trust.*

Personalization may also be associated with a competing risk-oriented evaluation. AI-enabled personalization relies on consumer data to generate individualized recommendations and may therefore be associated with greater awareness of data collection and inference and with heightened privacy concern (Lim & Kim, 2025). Because privacy concern is hypothesized to show a negative conditional association with purchase intention within the proposed structural model (Xie et al., 2020), personalization may show positive indirect associations through usefulness and trust while also showing an opposing negative indirect association through privacy concern. H8 therefore specifies the privacy-concern component of this competing pattern of indirect associations. Accordingly:

**H8.**  *Perceived personalization is expected to show a negative indirect association with purchase intention specifically through privacy concern.*

### 3.5. The Moderating Role of AI Literacy

AI literacy may influence how strongly psychological evaluations are associated with purchase intention, but this role is unlikely to be uniform across different evaluations. Greater AI literacy does not necessarily increase receptivity to AI and may instead encourage more critical and selective judgments (Tully et al., 2025). Its moderating role may therefore depend on the nature of the psychological evaluation involved.

Perceived usefulness is an instrumental and outcome-oriented evaluation with relatively direct relevance to purchase intention. Once consumers perceive that conversational AI improves search, comparison, or decision quality, the connection between this evaluation and purchase intention is already relatively clear. The strength of this association may therefore vary little across levels of AI literacy. Accordingly:

**H9a.**  *AI literacy is not expected to meaningfully alter the positive association between perceived usefulness and purchase intention, as evaluated relative to a specified study-specific practical-equivalence bound.*

Algorithmic trust involves greater evaluation-to-intention ambiguity because trusting an AI system does not necessarily translate directly into purchase intention. Trust in AI is associated with reliance on AI-generated advice (Klingbeil et al., 2024), while AI literacy can help consumers evaluate recommendations more selectively and calibrate their reliance according to AI capabilities and limitations (Leffrang et al., 2026). Consumers with greater AI literacy may therefore have a clearer basis for determining when trust is reflected in purchase intention. Accordingly:

**H9b.**  *AI literacy positively moderates the relationship between algorithmic trust and purchase intention, such that the positive relationship is stronger among consumers with higher AI literacy.*

Privacy concern similarly involves the extent to which a risk-related evaluation is reflected in purchase intention. Privacy concerns can discourage online participation and information disclosure, while privacy literacy can facilitate privacy-protective behavior (Baruh et al., 2017). Greater AI literacy may help consumers better recognize the practical implications of data collection, inference, and secondary use, which may be associated with a stronger negative relationship between privacy concern and purchase intention. Accordingly:

**H9c.**  *AI literacy negatively moderates the relationship between privacy concern and purchase intention, such that the negative relationship is stronger among consumers with higher AI literacy.*

## 4. Research Model and Method

### 4.1. Research Model and Design

**Figure 1:**
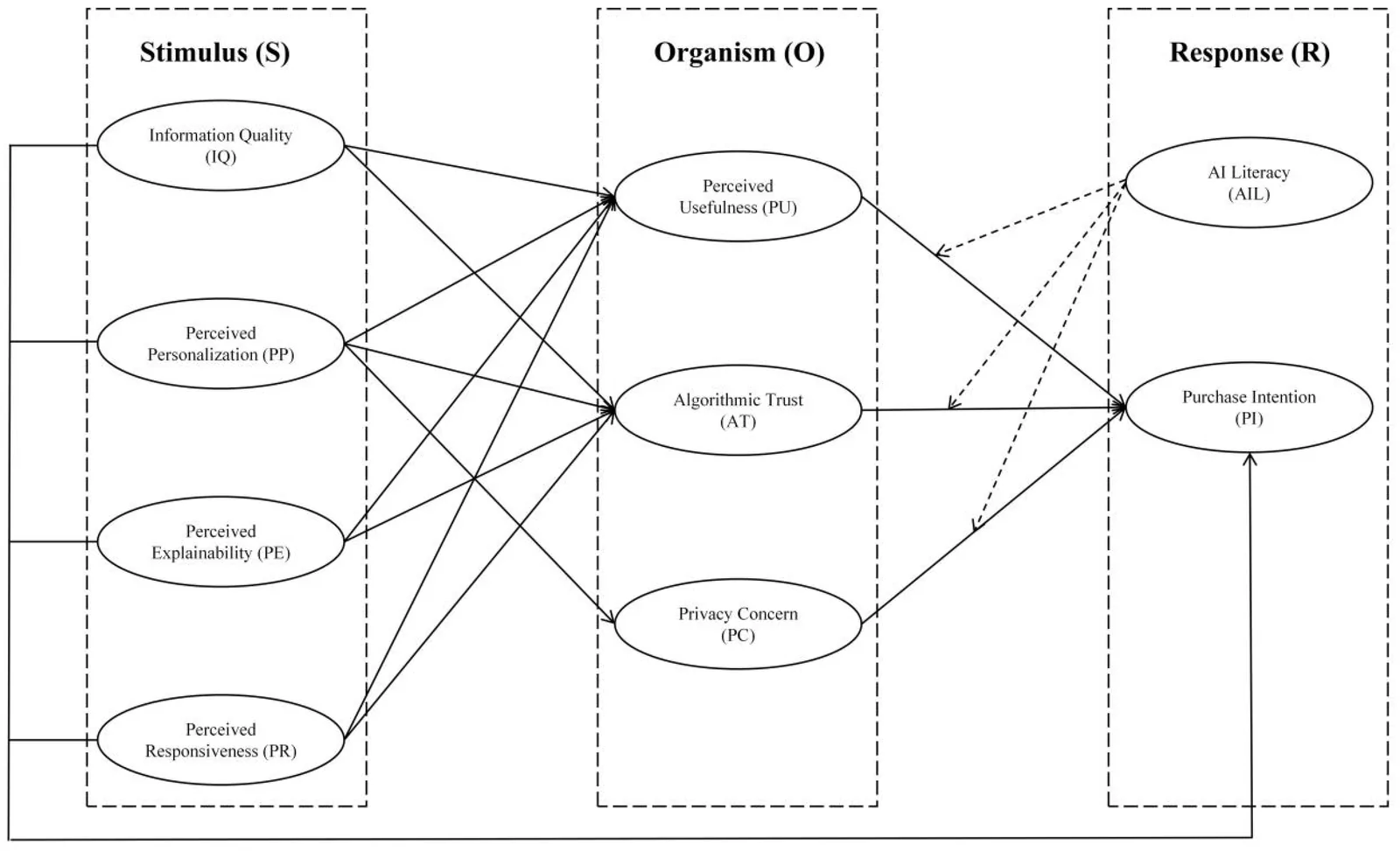
Proposed research model.

This study used a cross-sectional online survey following a standardized AI-assisted shopping task to examine how consumers respond to conversational AI shopping assistants. Based on the extended S-O-R framework, information quality (IQ), perceived personalization (PP), perceived explainability (PE), and perceived responsiveness (PR) were modeled as consumer-perceived conversational-AI cues; perceived usefulness (PU), algorithmic trust (AT), and privacy concern (PC) as parallel organismic evaluations; and purchase intention (PI) as the outcome variable. AI literacy (AIL) was introduced as a moderator of the relationships between the three organismic states and purchase intention. The four focal AI-related cues were measured as participants’ perceptions of their interaction experience and were not experimentally manipulated. Figure 1 presents the proposed research model.

**Figure 2:**
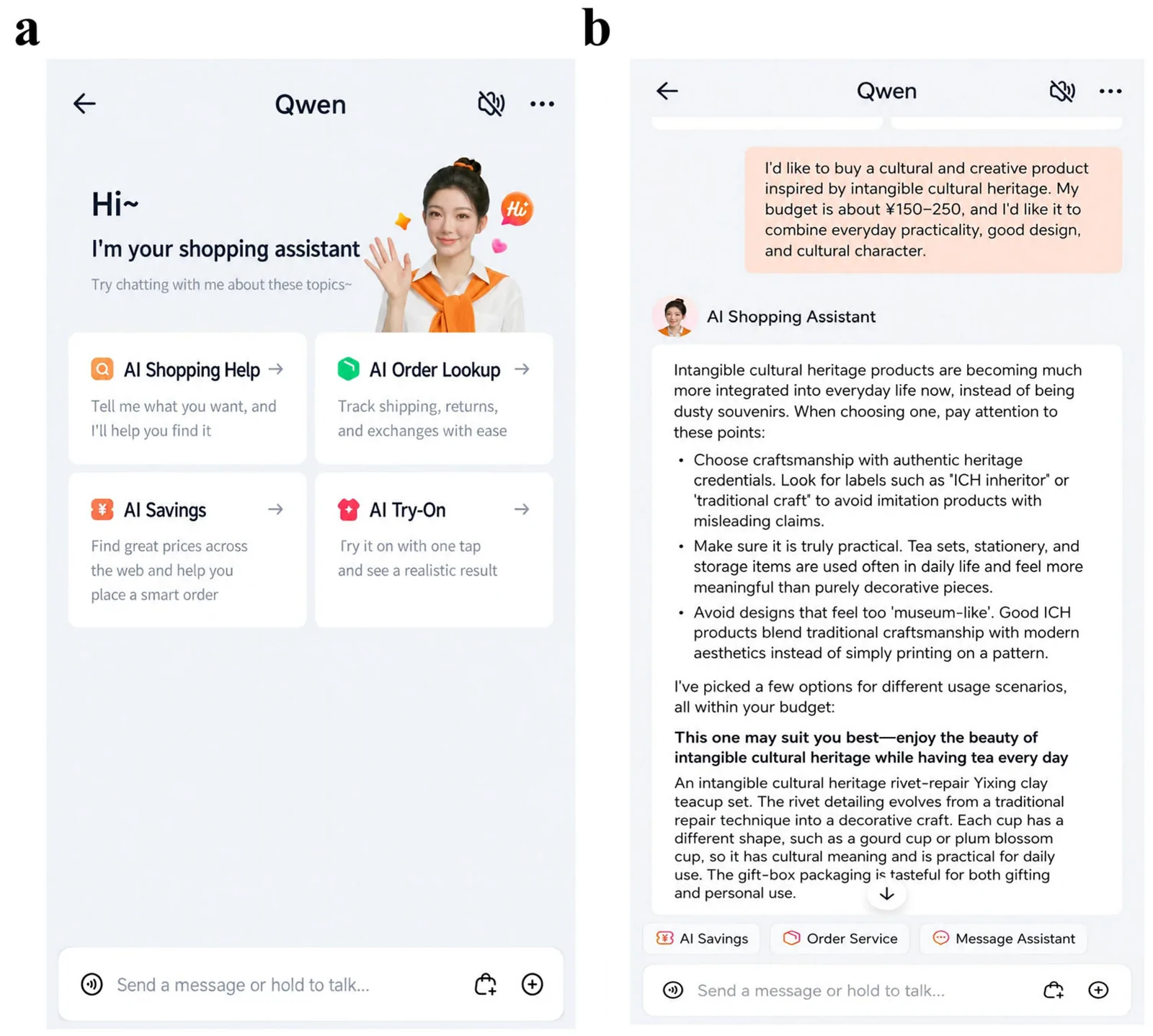
Taobao AI Shopping Assistant and Product Recommendation Scenario. Note. (**a**) Interface and functions of the Taobao AI Shopping Assistant. (**b**) AI-generated product recommendations based on a request for intangible cultural heritage (ICH) creative products priced at RMB 150–250, with an emphasis on practicality, design, and cultural characteristics.

To provide respondents with a common decision context, the study used a standardized shopping task involving the Taobao AI Shopping Assistant. Participants were instructed to use the assistant to search for intangible cultural heritage (ICH) creative products within a price range of RMB 150–250, with particular attention to practicality, design, and cultural characteristics. Rather than merely viewing or imagining a preconstructed interaction scenario, participants personally interacted with the AI shopping assistant to complete the assigned shopping task. They could engage in multi-turn exchanges, progressively refine their shopping criteria, and receive AI-generated product recommendations based on their requests (Figure 2). Thus, the task instructions and shopping objectives were standardized across participants, whereas the specific conversational exchanges and recommendations could vary according to participants’ interactions with the AI assistant. After completing the AI-assisted shopping task, participants proceeded to the questionnaire and evaluated the AI shopping assistant based on their interaction experience.

Importantly, information quality, perceived personalization, perceived explainability, and perceived responsiveness were not experimentally manipulated. The observed variation in these constructs therefore reflects between-participant differences in perceptions of the AI-supported interaction rather than experimentally induced differences in objective system features. Accordingly, these constructs are interpreted as consumer-perceived conversational-AI cues rather than manipulated technological stimuli.

### 4.2. Measurement

The questionnaire consisted of three sections. The first introduced the study and informed respondents that participation was voluntary and anonymous and that the collected data would be used solely for academic research. The second collected demographic characteristics and information on online-shopping behavior. The third measured the nine focal constructs in the proposed model. All items were assessed using a seven-point Likert scale ranging from 1 (strongly disagree) to 7 (strongly agree). Although voluntary and anonymous participation may reduce evaluation apprehension and response concerns, the use of the same respondents and response format for all focal constructs creates a potential risk of common method variance; this issue was therefore further assessed statistically.

The measurement items were developed with reference to established scales and prior operationalizations and were adapted to the conversational AI shopping context. AI literacy was measured with reference to established AI literacy measures (Carolus et al., 2023; Laupichler et al., 2023). The five items assessed self-perceived understanding of generative AI, effective prompting, evaluation of AI-generated information, identification of potential errors, and awareness of data use, algorithmic bias, and commercial influence. Consistent with the overarching conceptualization outlined in Section 2.5, these complementary competencies were modeled as indicators of a single AI literacy construct. Importantly, awareness of data-related issues is conceptually distinct from privacy concern: the former captures perceived ability to identify relevant issues, whereas the latter captures concern about the collection, inference, and use of personal information. Information quality drew on prior measures of information and chatbot service quality (Q. Chen et al., 2022; McKinney et al., 2002). Perceived personalization was informed by research on personalization in recommendation agents (Komiak & Benbasat, 2006), while perceived explainability was adapted from prior research on explainability and explanation facilities (Hamm et al., 2023; Wang & Benbasat, 2007). Perceived responsiveness was informed by prior research on responsiveness and AI-chatbot service quality (Q. Chen et al., 2022; J. Li et al., 2023).

Perceived usefulness was adapted from established technology-acceptance measures (Davis, 1989), while purchase intention drew on prior e-commerce and chatbot purchase-intention measures (Pavlou, 2003; Song & Shin, 2024). Algorithmic trust was informed by prior measures of trust in automated and recommendation systems (Jian et al., 2000; Wang & Benbasat, 2007), whereas privacy concern drew on established information-privacy measures (Malhotra et al., 2004; Smith et al., 1996). All items were reworded where necessary to fit the standardized conversational AI shopping scenario. The questionnaire was administered in Chinese. The measurement items were translated from English into Chinese and subsequently back-translated into English to assess the consistency of meaning between the original and translated versions. The complete measurement items are presented in Table 1.

### 4.3. Data Collection and Sample

Before the formal survey, a pretest with 30 participants assessed the clarity of the shopping scenario, the questionnaire wording, and the survey procedure. Minor revisions were made on the basis of participant feedback.

The formal survey was administered through Wenjuanxing, a widely used online survey platform in China. Participants were recruited through open online distribution of the survey invitation via WeChat Moments, WeChat groups, and QQ groups. Participation was voluntary, and no monetary or other material incentives were provided. Because the survey link was openly distributed through social-media channels rather than sent to a fixed sampling frame, the number of individuals who viewed the invitation but chose not to participate could not be determined; therefore, a conventional response rate could not be calculated. Two screening criteria were applied at the beginning of the questionnaire: respondents were required to be at least 18 years old and to have prior online-shopping experience. Following the eligibility screening, 636 questionnaires were submitted. Of these, 28 were excluded because the questionnaire was completed in less than two minutes, and 20 were excluded because they exhibited identical or mechanical response patterns indicative of insufficient response quality. After data screening, 588 valid questionnaires were retained for subsequent analysis, representing 92.45% of the 636 submitted questionnaires.

The final sample consisted of 43.54% male and 56.46% female participants. Participants aged 26–45 years accounted for 68.37% of the sample, and 71.94% held a bachelor’s degree or above. In addition, 89.29% reported shopping online at least several times per month, indicating substantial familiarity with online shopping environments. Detailed distributions of gender, age, education, monthly income, and online-shopping frequency are reported in Appendix A (Table A1).

### 4.4. Data Analysis

Data screening and preliminary statistical analyses were conducted with IBM SPSS Statistics 27.0, and covariance-based structural equation modeling (CB-SEM) was performed with IBM SPSS Amos 29.0. CB-SEM was used to test the theoretically specified relationships among multiple latent constructs. Following the recommended two-step approach, the measurement model was evaluated before testing the structural model (Anderson & Gerbing, 1988).

First, confirmatory factor analysis (CFA) evaluated the measurement model. Internal consistency was assessed using Cronbach’s alpha and composite reliability (CR), and convergent validity was evaluated through standardized factor loadings and average variance extracted (AVE) (Fornell & Larcker, 1981). Discriminant validity was assessed using both the Fornell–Larcker criterion and heterotrait–monotrait (HTMT) ratios (Voorhees et al., 2016). Overall model fit was evaluated using multiple absolute and incremental fit indices (Hu & Bentler, 1999). Given that the focal constructs were measured contemporaneously from the same respondents using the same response format, a CFA-based common latent factor (CLF) analysis was additionally conducted as a robustness check for potential common method variance.

Second, the structural model was estimated to test the hypotheses. Standardized path coefficients were used to evaluate the direct relationships. Direct, indirect, and total effects were assessed using bias-corrected bootstrap confidence intervals (Preacher & Hayes, 2008). The moderating role of AI literacy was examined separately using an observed-score analysis in IBM SPSS Statistics 27.0 with Hayes’ PROCESS macro (Version 5.0, Model 14), rather than through latent-variable interactions in AMOS. Perceived usefulness (PU), algorithmic trust (AT), and privacy concern (PC) were specified as parallel mediators, and AI literacy (AIL) was specified as a moderator of the associations between these organismic states and purchase intention (PI). In the reported PROCESS specification, information quality was entered as the focal predictor, while perceived personalization, perceived explainability, and perceived responsiveness were included as covariates. The observed composite scores used in the PROCESS analysis were calculated by averaging the corresponding items for each construct. PU, AT, PC, and AIL were mean-centered before the interaction terms were constructed. Significant interactions were probed using simple-slope analyses at low (−1 SD), mean, and high (+1 SD) levels of AI literacy (Dawson, 2014). For each moderated association, the conditional slope was calculated as b_1_ + b_3_W, where b_1_ represents the coefficient of the focal psychological evaluation, b_3_ represents its interaction coefficient with AI literacy, and W represents the specified centered value of AI literacy. Ninety-five percent confidence intervals were used for statistical inference.

## 5. Results

### 5.1. Measurement Model

The measurement model showed satisfactory reliability and convergent validity. Standardized factor loadings ranged from 0.683 to 0.840. Cronbach’s alpha and composite reliability (CR) both ranged from 0.844 to 0.867, above commonly accepted thresholds. Average variance extracted (AVE) values ranged from 0.567 to 0.602, supporting convergent validity. Table 2 reports the detailed results.

The Kaiser–Meyer–Olkin (KMO) value was 0.943, and Bartlett’s test of sphericity was significant (χ^2^ = 11,438.016, df = 666, *p* < 0.001), confirming that the data were suitable for factor analysis (Table 3). Discriminant validity was also supported. As Table 4 shows, the square root of the AVE for each construct exceeded its correlations with other constructs, and all heterotrait–monotrait (HTMT) ratios remained below 0.70 (Table 5), indicating adequate empirical distinction among the nine constructs.

The confirmatory factor analysis showed satisfactory overall fit (Table 6): CMIN/DF = 1.307, RMSEA = 0.023, GFI = 0.936, AGFI = 0.924, NFI = 0.934, TLI = 0.981, IFI = 0.984, and CFI = 0.983. The parsimonious indices also met the recommended criteria (PCFI = 0.876, PNFI = 0.831, PGFI = 0.789). These results supported the adequacy of the measurement model and justified proceeding to structural model testing.

Because the focal constructs were measured contemporaneously from the same respondents using the same response format, we conducted a CFA-based common latent factor (CLF) analysis as a supplementary assessment of potential common method variance. A common latent factor was added to the baseline CFA measurement model, with all observed items additionally loading on the common factor. The CLF specification produced only negligible changes in model fit relative to the baseline CFA model: CFI increased from 0.983 to 0.984 (ΔCFI = 0.001), while RMSEA decreased from 0.023 to 0.022 (ΔRMSEA = −0.001). Thus, the supplementary analysis provided no evidence that adding a general common-method factor materially improved model fit. Nevertheless, this result should not be interpreted as ruling out all forms of common method variance, given the same-source and contemporaneous self-report design.

### 5.2. Structural Model and Hypothesis Testing

The structural coefficients reported below are interpreted as conditional associations within the specified model. Given the cross-sectional design and contemporaneous measurement of the focal constructs, these coefficients should not be interpreted as establishing causal relationships or temporal ordering among the constructs.

The structural model also demonstrated satisfactory fit (CMIN/DF = 1.430, RMSEA = 0.027, GFI = 0.938, AGFI = 0.926, NFI = 0.937, RFI = 0.929, TLI = 0.978, IFI = 0.980, and CFI = 0.980). The parsimonious fit indices were also acceptable (PCFI = 0.873, PNFI = 0.835, and PGFI = 0.785).

**Figure 3:**
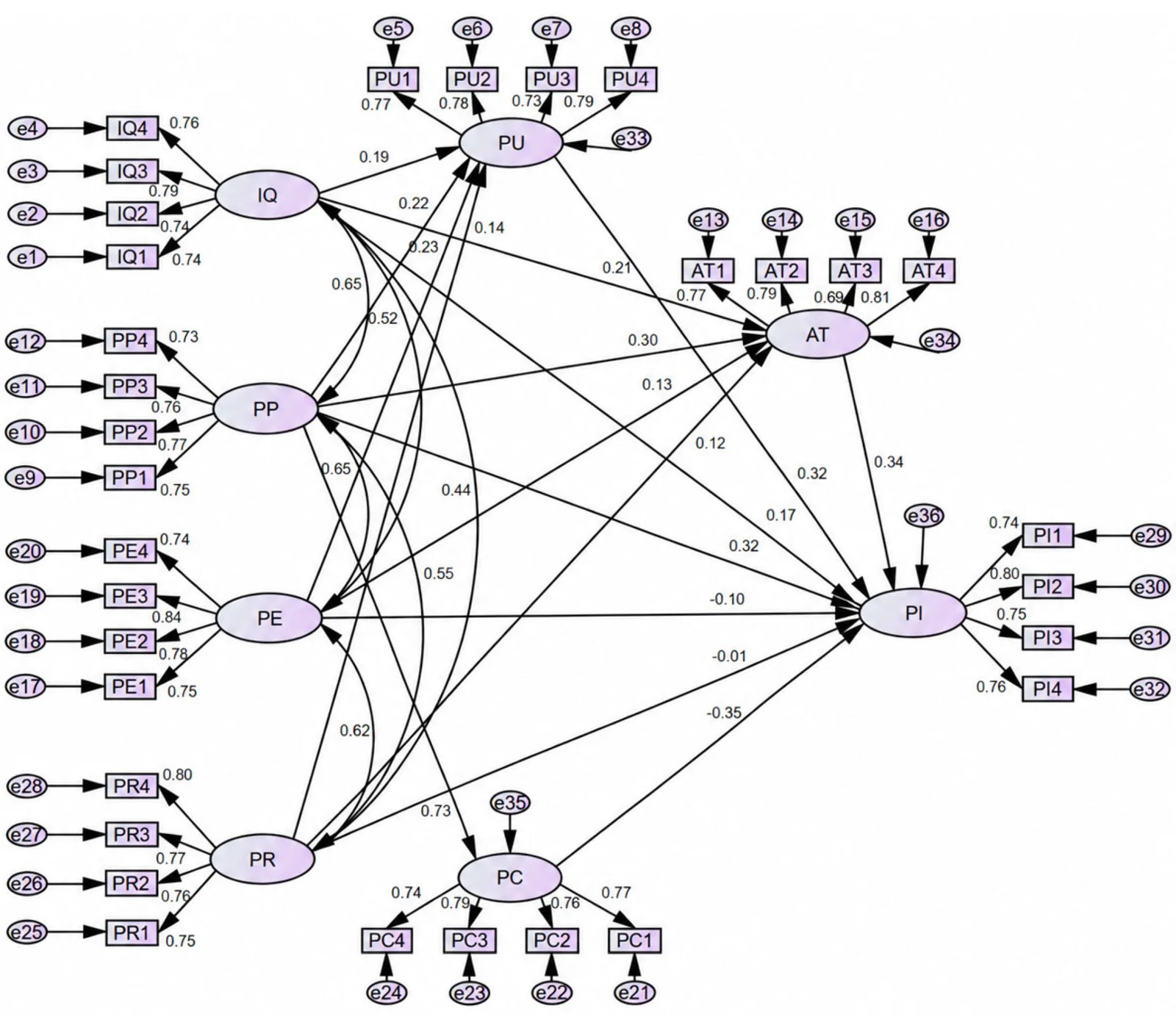
Standardized structural model results.

Figure 3 presents the standardized structural model, and Table 7 summarizes the direct-effect hypothesis tests. All four consumer-perceived conversational-AI cues were positively associated with perceived usefulness: information quality (β = 0.189, *p* = 0.001), perceived personalization (β = 0.218, *p* = 0.001), perceived explainability (β = 0.228, *p* < 0.001), and perceived responsiveness (β = 0.144, *p* = 0.011), supporting H1a–H1d. Perceived explainability showed the strongest association with perceived usefulness.

The four characteristics also were positively associated with algorithmic trust: information quality (β = 0.210, *p* < 0.001), perceived personalization (β = 0.303, *p* < 0.001), perceived explainability (β = 0.134, *p* = 0.038), and perceived responsiveness (β = 0.118, *p* = 0.036), supporting H2a–H2d. Perceived personalization had the largest positive structural coefficient for algorithmic trust. Personalization also was positively associated with privacy concern (β = 0.730, *p* < 0.001), supporting H3 and indicating that it is simultaneously associated with benefit- and risk-oriented psychological responses.

All three organismic states showed significant conditional associations with purchase intention in the structural model. Perceived usefulness (β = 0.322, *p* < 0.001) and algorithmic trust (β = 0.336, *p* < 0.001) were positively associated with purchase intention, while privacy concern showed a significant negative conditional association (β = −0.350, *p* < 0.001). H4a–H4c were therefore supported within the specified structural model. Notably, the negative structural coefficient for privacy concern differs in sign from its positive zero-order correlation with purchase intention (r = 0.236; Table 4). This sign reversal was therefore examined in supplementary sequential and collinearity analyses reported below.

Because privacy concern showed a positive zero-order association with purchase intention (r = 0.236) but a negative structural coefficient in the full SEM (β = −0.350, *p* < 0.001), supplementary analyses were conducted to examine this sign reversal. The standardized PC → PI coefficient was 0.236 when privacy concern was considered alone, decreased to 0.030 after perceived usefulness was included, and changed sign to −0.107 after algorithmic trust was additionally introduced. With perceived personalization incorporated, the coefficient further decreased to −0.308, while the coefficient in the full latent-variable SEM was −0.350 (*p* < 0.001). This progressive change is consistent with a suppression pattern, in which the positive bivariate association is attenuated and reversed as overlapping variance with other predictors is statistically controlled (Appendix A, Table A2).

Collinearity diagnostics using composite scores provided no indication of problematic multicollinearity. Tolerance values ranged from 0.514 to 0.672, while VIF values ranged from 1.488 to 1.944. For privacy concern specifically, the tolerance value was 0.631 and the VIF was 1.585 (Appendix A, Table A3). In a supplementary composite-score regression including all seven predictors, privacy concern remained negatively associated with purchase intention (β = −0.218, t = −5.187, *p* < 0.001). Accordingly, the negative PC → PI coefficient in the full SEM is interpreted as a conditional structural association after accounting for shared variance with other predictors, rather than as a negative zero-order or causal relationship.

Alternative Model Analysis. To examine an alternative structural specification that includes a relationship from perceived usefulness (PU) to algorithmic trust (AT), we estimated two additional models. Model 2 added a PU → AT path to the focal parallel model while retaining the direct paths from the four consumer-perceived conversational-AI cues to AT. The added PU → AT path was not statistically significant (β = 0.095, *p* = 0.087), and Model 2 did not significantly improve fit relative to Model 1, Δχ^2^(1) = 2.920, *p* = 0.087. The overall fit indices were also essentially unchanged (Model 1: CFI = 0.980, TLI = 0.978, RMSEA = 0.027, AIC = 803.874; Model 2: CFI = 0.980, TLI = 0.978, RMSEA = 0.027, AIC = 802.954).

Model 3 represented a more restrictive sequential specification in which PU → AT was retained while the four direct cue → AT paths were constrained to zero. Because Model 3 is nested within Model 2, these two specifications were compared using a chi-square difference test. Constraining the four cue → AT paths to zero significantly worsened model fit relative to Model 2, Δχ^2^(4) = 107.002, *p* < 0.001. Model 3 also showed poorer global fit (χ^2^(445) = 735.956, CFI = 0.969, TLI = 0.966, RMSEA = 0.033, AIC = 901.956) than the focal parallel model. Taken together, these comparisons support retaining the parallel specification as the focal model. However, because PU and AT were measured contemporaneously in a cross-sectional design, these results should not be interpreted as establishing their temporal or causal ordering. The alternative-model comparison is reported in Appendix A (Table A4).

The direct associations of the four consumer-perceived conversational-AI cues followed a differentiated pattern. Information quality (β = 0.169, *p* = 0.005) and perceived personalization (β = 0.324, *p* < 0.001) retained significant positive direct associations with purchase intention, supporting H5a and H5b. In contrast, the residual direct coefficients for perceived explainability (β = −0.096, *p* = 0.141) and perceived responsiveness (β = −0.008, *p* = 0.891) were not statistically significant. These nonsignificant coefficients are not interpreted as evidence that the residual direct associations are zero. Because H5c and H5d concern total indirect associations rather than residual direct paths, they are evaluated separately in Section 5.3.

Because differences in individual significance do not establish differences between coefficients, supplementary equality-constraint tests were conducted. Constraining the IQ → PI and PE → PI paths to equality significantly worsened model fit, Δχ^2^(1) = 8.564, *p* = 0.003. Constraining the PP → PI and PR → PI paths to equality also significantly worsened model fit, Δχ^2^(1) = 10.098, *p* = 0.001. These tests indicate that the corresponding structural regression weights differ in the present model. The equality-constraint tests are summarized in Appendix A (Table A5).

### 5.3. Mediation Analysis

The bootstrap estimates reported below are interpreted as statistical indirect associations within the specified structural model. Because the antecedents, proposed mediators, and purchase intention were measured contemporaneously, these estimates should not be interpreted as establishing temporal or causal mediation. Rather, they indicate patterns that are consistent with the proposed mediation structure.

The bootstrap analysis, based on 5000 bias-corrected bootstrap samples, yielded indirect-association patterns consistent with the proposed parallel benefit–risk structure (Table 8). The standardized total indirect associations of information quality (β = 0.131, *p* < 0.001), perceived explainability (β = 0.118, *p* = 0.001), and perceived responsiveness (β = 0.086, *p* = 0.004) with purchase intention were positive and significant. The significant positive total indirect associations of perceived explainability and perceived responsiveness were consistent with H5c and H5d, respectively. The aggregate indirect association of perceived personalization, by contrast, was not significant (β = −0.084, *p* = 0.195), reflecting indirect pathways operating in opposing directions.

The specific indirect estimates provide a clearer view of these model-implied associations (Table 9). Significant positive indirect associations with purchase intention through perceived usefulness were observed for information quality (β = 0.061, *p* < 0.001), personalization (β = 0.070, *p* = 0.001), explainability (β = 0.073, *p* < 0.001), and responsiveness (β = 0.046, *p* = 0.010), consistent with H6a–H6d. Significant positive indirect associations through algorithmic trust were also observed for information quality (β = 0.070, *p* < 0.001), personalization (β = 0.102, *p* < 0.001), explainability (β = 0.045, *p* = 0.033), and responsiveness (β = 0.040, *p* = 0.039), consistent with H7a–H7d.

Personalization also showed a significant negative indirect association with purchase intention through privacy concern (β = −0.255, *p* < 0.001), consistent with H8.

Personalization showed positive indirect associations through perceived usefulness and algorithmic trust and a negative indirect association through privacy concern at the same time. Perceived personalization also showed a significant positive direct association with purchase intention (β = 0.324, *p* < 0.001), while the total association remained positive and statistically significant (β = 0.241, *p* = 0.002). This pattern indicates that the indirect pathways operate in opposing directions. In particular, the negative privacy-related pathway may attenuate some of the positive association linked to personalization, but it does not eliminate the overall positive association.

### 5.4. Results of the AI Literacy Moderation Analysis

**Figure 4:**
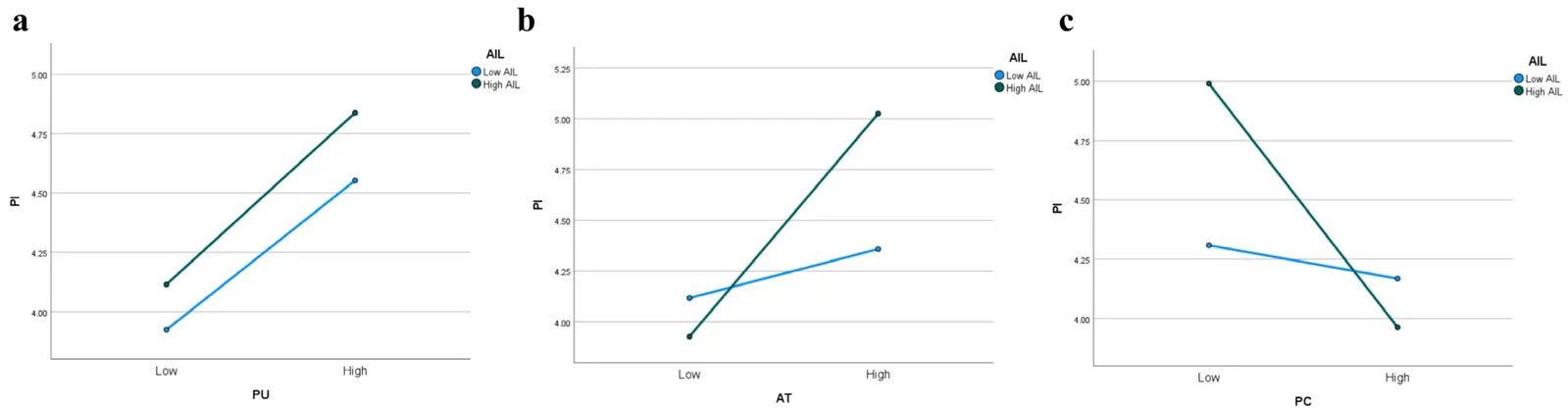
Interaction plots for the moderating role of AI literacy in the associations between (**a**) perceived usefulness and purchase intention, (**b**) algorithmic trust and purchase intention, and (**c**) privacy concern and purchase intention. Low and high AI literacy represent values at 1 SD below and 1 SD above the mean, respectively.

Table 10 reports the moderating associations of AI literacy, and Figure 4 illustrates these relationships. The PU × AI literacy interaction was not statistically significant (B = 0.0142, SE = 0.0299, *p* = 0.6345, 95% CI [−0.0444, 0.0728]). Because a nonsignificant interaction alone cannot establish that the usefulness–purchase intention association is stable, a supplementary equivalence analysis was conducted for H9a.

For this supplementary analysis, a study-specific practical-equivalence interval of B = [−0.10, 0.10] was specified. Under the metric used in the analysis, this interval represents a change of less than 0.10 in the PU–PI slope for each one-unit change in AI literacy. The ±0.10 bound is treated as a study-specific practical-equivalence criterion for this supplementary analysis and is not presented as a universally accepted small-effect threshold in behavioral research. The two one-sided tests indicated that the observed interaction coefficient fell within the specified equivalence bounds (lower-bound test: t = 3.82, *p* < 0.001; upper-bound test: t = −2.87, *p* < 0.01). Thus, the observed interaction is statistically compatible with practical equivalence relative to the specified ±0.10 bound, providing supplementary evidence relevant to H9a under this study-specific criterion. The supplementary equivalence analysis is summarized in Appendix A (Table A6).

AI literacy significantly strengthened the positive relationship between algorithmic trust and purchase intention (B = 0.1307, *p* < 0.001, 95% CI [0.0714, 0.1900]), supporting H9b; the positive association between algorithmic trust and purchase intention was stronger among consumers with higher AI literacy. AI literacy also moderated the relationship between privacy concern and purchase intention in the predicted negative direction (B = −0.1330, *p* < 0.001, 95% CI [−0.1859, −0.0801]), supporting H9c. Privacy concern therefore had a stronger negative association with purchase intention among consumers with higher AI literacy.

Simple-slope analyses were subsequently conducted to probe the two statistically significant interactions. For H9b, the conditional positive association between algorithmic trust and purchase intention became progressively stronger as AI literacy increased. At low AI literacy (−1 SD), the conditional association was B = 0.0934 (SE = 0.0564, *p* = 0.0985, 95% CI [−0.0175, 0.2042]). At the mean level of AI literacy, the association was positive and statistically significant (B = 0.2591, SE = 0.0394, *p* < 0.001, 95% CI [0.1817, 0.3365]). At high AI literacy (+1 SD), the positive association was stronger (B = 0.4249, SE = 0.0534, *p* < 0.001, 95% CI [0.3200, 0.5298]). Thus, the conditional AT–PI association was not statistically significant at low AI literacy but was positive and statistically significant at the mean and high levels of AI literacy.

For H9c, the conditional association between privacy concern and purchase intention became increasingly negative as AI literacy increased. At low AI literacy (−1 SD), the conditional association was B = −0.0534 (SE = 0.0532, *p* = 0.3157, 95% CI [−0.1579, 0.0511]). At the mean level of AI literacy, the association was negative and statistically significant (B = −0.2221, SE = 0.0425, *p* < 0.001, 95% CI [−0.3056, −0.1386]). At high AI literacy (+1 SD), the negative association was stronger (B = −0.3907, SE = 0.0558, *p* < 0.001, 95% CI [−0.5003, −0.2812]). Thus, the conditional PC–PI association was not statistically significant at low AI literacy but was negative and statistically significant at the mean and high levels of AI literacy.

Taken together, the moderation and simple-slope results show an asymmetric pattern. The AT–PI association became progressively more positive as AI literacy increased, whereas the PC–PI association became progressively more negative. In contrast, the PU × AI literacy interaction was statistically compatible with practical equivalence relative to the specified study-specific ±0.10 bound. This pattern is consistent with an evaluation-to-intention perspective in which the moderating role of AI literacy differs across psychological evaluations rather than uniformly strengthening their associations with purchase intention.

## 6. Discussion

This study examined purchase-intention formation in conversational-AI shopping through parallel benefit- and risk-oriented psychological evaluations. Given the cross-sectional design, the structural and indirect relationships discussed below are interpreted as associations consistent with the proposed theoretical framework rather than as evidence of temporal or causal psychological mechanisms. The findings indicate that the observed associations do not reflect a uniformly positive acceptance pattern. Information quality, personalization, explainability, and responsiveness were positively associated with perceived usefulness and algorithmic trust, while personalization was also positively associated with privacy concern. These psychological evaluations showed distinct conditional associations with purchase intention. Consumer responses to conversational AI are therefore better understood in terms of the joint associations of instrumental value, trust, and perceived privacy risk with purchase intention.

The findings should also be interpreted in light of the common measurement source. Although the supplementary CLF analysis did not indicate that adding a general common-method factor materially improved model fit, all focal constructs were assessed contemporaneously from the same respondents using the same response format. Shared method variance therefore cannot be ruled out completely and may contribute to covariance among the self-reported constructs.

### 6.1. Theoretical Implications

First, the findings indicate that conversational AI characteristics show differentiated associations with the psychological evaluations and purchase intention examined in the model. Explainability showed the strongest association with perceived usefulness, suggesting that understandable explanations create instrumental value by helping consumers evaluate AI-generated recommendations. This is consistent with research showing that explanations can enhance transparency, interpretability, and trust in conversational recommendations (Q. Q. Chen et al., 2025). Personalization showed the strongest association with algorithmic trust, while information quality contributed significantly to both usefulness and trust. These results suggest that AI characteristics provide distinct psychological cues rather than functioning as interchangeable indicators of system quality.

Second, personalization reveals an important pattern of competing associations in AI-assisted consumption. Greater personalization was positively associated with perceived usefulness and algorithmic trust while also being positively associated with privacy concern. Its positive indirect associations through usefulness and trust coexisted with a negative indirect association through privacy concern, resulting in a nonsignificant aggregate indirect association. However, the negative privacy-related pathway should be interpreted as a conditional association within the multivariable structural model. Privacy concern was positively correlated with purchase intention at the zero-order level (r = 0.236) but showed a negative conditional association in the full structural model (β = −0.350, *p* < 0.001), a sign reversal that the supplementary analyses indicated was consistent with a suppression pattern. Similar evidence suggests that personalized AI communication can generate competing perceptions of helpfulness and intrusiveness (Zobair et al., 2026). Personalization should therefore be understood as being associated with competing benefit- and privacy-related evaluations rather than as producing a uniformly positive or negative response. Importantly, despite the negative privacy-related indirect association, personalization retained a significant positive direct association with purchase intention (β = 0.324, *p* < 0.001), and its total association remained positive and significant (β = 0.241, *p* = 0.002). Thus, the privacy-related pathway may attenuate, but does not eliminate, the overall positive association between personalization and purchase intention. The remaining positive direct association may reflect aspects of personalized decision support not fully represented by perceived usefulness, algorithmic trust, and privacy concern. From a content-cue perspective, personalization may be associated with the relevance of recommended products and the composition of the alternatives consumers consider. These content-related aspects are conceptually distinct from general perceptions of usefulness, trust in AI-generated recommendations, and concerns about personal information. They therefore offer a possible explanation for the residual personalization–purchase intention association observed after accounting for the three psychological evaluations. Because such processes were not directly measured, they should be examined in future research rather than treated as established mechanisms.

Third, the findings are consistent with the distinction between content-related and interaction-process cues. Information quality and personalization retained significant positive direct associations with purchase intention, whereas explainability and responsiveness showed significant positive indirect associations through perceived usefulness and algorithmic trust, while their residual direct coefficients were not statistically significant. These nonsignificant coefficients, however, should not be interpreted as evidence that the residual direct associations are zero or that full or near-full mediation has been established. Supplementary equality-constraint tests further indicated significant differences between the IQ → PI and PE → PI coefficients and between the PP → PI and PR → PI coefficients. Thus, within the specified cross-sectional model, the associations of explainability and responsiveness with purchase intention were more evident in the estimated indirect component through perceived usefulness and algorithmic trust than in their residual direct coefficients. This pattern is consistent with evidence that different chatbot characteristics can be associated with consumer responses through different psychological evaluations (Yang et al., 2026). Because the focal variables were measured contemporaneously, however, these patterns should be interpreted as structural associations consistent with the proposed content–process distinction rather than as evidence of distinct causal psychological mechanisms.

Fourth, the moderation results provide a more differentiated understanding of AI literacy. The PU × AI literacy interaction was not statistically significant, while the supplementary equivalence analysis indicated that the observed interaction was statistically compatible with practical equivalence relative to the specified study-specific ±0.10 bound. This finding should therefore be interpreted relative to the adopted equivalence criterion rather than as evidence that AI literacy has no moderating association with the usefulness–intention relationship. In contrast, AI literacy significantly moderated both the algorithmic trust–purchase intention and privacy concern–purchase intention relationships, with the former interaction being positive and the latter negative. This asymmetric pattern suggests that the moderating role of AI literacy may differ across psychological evaluations rather than uniformly strengthening their associations with purchase intention. Prior research likewise indicates that the consequences of AI literacy can vary across psychological pathways (Goyanes et al., 2026). Within the present model, higher AI literacy was associated with a stronger positive relationship between algorithmic trust and purchase intention and a stronger negative conditional relationship between privacy concern and purchase intention. These patterns are consistent with an evaluation-to-intention perspective, although the cross-sectional design does not establish a psychological process linking evaluations to subsequent behavior.

Taken together, these findings extend the S-O-R perspective by showing that consumer-perceived conversational-AI cues on the stimulus side of the framework are associated with coexisting and potentially competing psychological evaluations, and that their associations with purchase intention may vary partly with consumers’ AI-related capabilities. The results therefore move beyond a simple technology-acceptance perspective toward an account in which consumers simultaneously evaluate the benefits and risks of AI and differ in how strongly these judgments are associated with purchase intention.

### 6.2. Managerial Implications

The findings also provide practical implications for retailers and developers of conversational AI shopping systems. First, information quality should remain a fundamental design priority because accurate, relevant, and consistent information contributes to usefulness, algorithmic trust, and purchase intention. Conversational sophistication cannot compensate for weak informational foundations.

Second, explainability and responsiveness should primarily support the consumer decision process. Explanations should clarify why products are recommended and how product attributes correspond to consumer needs, while responsive dialogue should allow consumers to refine preferences and resolve uncertainty efficiently. Their value lies less in directly increasing product attractiveness than in making AI-supported decisions easier to understand and trust.

Third, personalization requires careful calibration. Although tailored recommendations can increase relevance and strengthen trust, highly salient or unexplained personalization may simultaneously heighten privacy concern. Retailers should therefore combine personalization with transparent data-use practices, appropriate limits on information collection, and meaningful consumer control. The objective should be to create sufficient personalization to improve decision value without making the interaction appear intrusive.

Finally, the moderating role of AI literacy suggests that the associations of algorithmic trust and privacy concern with purchase intention may be especially pronounced among consumers with higher AI literacy. For these consumers, clear communication about system capabilities, recommendation logic, and data practices may be particularly important because trust and privacy concern are more strongly associated with stated purchase intention. Conversational AI design should therefore support not only effective recommendations but also consumers’ ability to understand why the system can be trusted and how their data are handled.

## 7. Conclusions

This study developed and tested an extended S-O-R framework to examine purchase-intention formation in conversational-AI shopping through parallel benefit- and risk-oriented psychological evaluations. Information quality, perceived personalization, perceived explainability, and perceived responsiveness were treated as consumer-perceived conversational-AI cues on the stimulus side of the framework; perceived usefulness, algorithmic trust, and privacy concern as parallel psychological states; and AI literacy as a boundary condition affecting how these evaluations are associated with purchase intention.

The findings reveal a pattern of competing benefit- and risk-oriented psychological evaluations in consumer responses to conversational AI. All four consumer-perceived conversational-AI cues were positively associated with perceived usefulness and algorithmic trust, while perceived personalization was also positively associated with privacy concern. Most notably, personalization showed competing indirect associations with purchase intention: positive indirect associations through perceived usefulness and algorithmic trust coexisted with a negative indirect association through privacy concern. The privacy-related pathway may therefore attenuate, but does not eliminate, the overall positive association between personalization and purchase intention.

The study further shows that AI literacy selectively moderates the associations of algorithmic trust and privacy concern with purchase intention, while the usefulness–intention interaction is statistically compatible with practical equivalence relative to the specified study-specific bound. This pattern is consistent with an evaluation-to-intention perspective, in which the associations between psychological evaluations and stated purchase intentions vary with AI literacy. Because the study measured purchase intention rather than observed purchasing behavior, these findings should not be interpreted as evidence that consumers acted more consistently with their evaluations.

### Limitations and Future Research

First, the cross-sectional design and contemporaneous measurement of the focal constructs preclude establishing temporal ordering or causal relationships among the variables. Accordingly, the structural and indirect associations identified in this study should not be interpreted as causal effects or as evidence of causal psychological mechanisms. Relatedly, information quality, perceived personalization, perceived explainability, and perceived responsiveness were measured as consumers’ perceptions of their AI interaction experience rather than as experimentally manipulated system features. Although participants directly interacted with the Taobao AI Shopping Assistant during a standardized shopping task, observed variation in these four constructs cannot be attributed to experimentally induced differences in objective system features. Future experimental and longitudinal studies could manipulate specific conversational AI characteristics, examine how consumer perceptions, evaluations, and purchase intentions evolve across repeated interactions, and incorporate behavioral outcomes such as recommendation acceptance, click-through behavior, transaction records, repeat purchases, or switching behavior. Such designs would help determine whether the observed evaluation–intention associations extend to actual consumer behavior.

Second, the same-source, contemporaneous self-report design creates a potential risk of common method variance. Although respondents were informed that participation was voluntary and anonymous and the supplementary CFA-based common latent factor analysis did not indicate a material improvement in model fit after adding a general common factor, these procedures do not eliminate all possible forms of shared method variance. Future research could reduce this concern through temporal separation of predictor and outcome measurements, multi-source data, behavioral outcome measures, or appropriately designed marker-variable approaches.

Third, the empirical setting was limited to Chinese online shoppers interacting with the Taobao AI Shopping Assistant in a standardized shopping task involving intangible cultural heritage creative products. Although the standardized task provided participants with a common interaction context, it did not control for pre-existing differences in familiarity with Taobao, trust in the platform, or platform-specific privacy perceptions, none of which were separately measured in the present study. These pre-existing platform evaluations may therefore have been associated with participants’ assessments of the AI shopping assistant, including algorithmic trust, privacy concern, and purchase intention. In addition, because recruitment relied on open social-media distribution, a conventional response rate could not be determined, and the absence of regional information and technical duplicate-submission screening limits a fuller assessment of sample coverage. The relative importance of usefulness, trust, and privacy concern may vary across platforms, product categories, and cultural environments. Future studies should therefore replicate the proposed model across different conversational-commerce platforms and directly measure or experimentally control prior platform familiarity, platform trust, and platform-specific privacy perceptions. Such designs would help distinguish associations related to consumers’ perceptions of the conversational AI interaction from those potentially associated with pre-existing evaluations of the underlying platform. Cross-platform and cross-cultural research would also strengthen the generalizability of the findings.

Fourth, AI literacy was measured through self-reported knowledge and competence. Although the present study operationalized AI literacy as a single overarching construct, its five items encompass distinguishable competencies, and the current measurement results do not establish that a unidimensional specification is superior to alternative multidimensional models. Future studies could combine subjective measures with objective knowledge tests or task-based assessments and distinguish dimensions such as AI knowledge, interaction skills, critical evaluation, and data-awareness competence. They could also examine whether data-awareness competence is empirically distinguishable from privacy-related knowledge and concern at a more fine-grained level. This may clarify which aspects of AI literacy are most relevant to trust-based reliance and privacy-sensitive decisions.

Finally, future research could examine nonlinear and dynamic benefit–risk relationships. Increasing personalization may initially enhance relevance and trust but eventually intensify privacy concern when consumers perceive that the system knows “too much.” Repeated interactions may also alter the balance between trust accumulation and privacy sensitivity over time. Examining these processes would provide a more dynamic account of consumer decision making with conversational AI.

## Figures and Tables

**Table 1:** Measurement items.

Construct	Measurement Item	References
AI Literacy (AIL)	I understand the basic way generative AI produces answers or suggestions based on user input.	(Carolus et al., 2023; Laupichler et al., 2023)
	I can obtain results that better match my needs by clearly expressing and adjusting my requests to AI.	
	I can judge whether the information or suggestions provided by AI are reasonable.	
	I can identify possible errors, outdated content, or misunderstandings in AI-generated information.	
	I can identify issues related to data use, algorithmic bias, or commercial influence when using AI.	
Information Quality (IQ)	The product information provided by the AI shopping assistant is generally accurate.	(Q. Chen et al., 2022; McKinney et al., 2002)
	The AI shopping assistant provides the key information needed to complete this product selection task.	
	The information provided by the AI shopping assistant is relevant to my shopping needs.	
	The information provided by the AI shopping assistant is generally consistent across different rounds of interaction.	
Perceived Personalization (PP)	The AI shopping assistant provides targeted recommendations based on my individual needs.	(Komiak & Benbasat, 2006)
	The AI shopping assistant’s recommendations reflect my budget, intended use, or preferences.	
	When I modify my shopping criteria, the AI shopping assistant adjusts its recommendations accordingly.	
	Compared with general product recommendations, the suggestions provided by the AI shopping assistant are clearly customized to me.	
Perceived Explainability (PE)	The AI shopping assistant clearly explains why these products are recommended.	(Hamm et al., 2023; Wang & Benbasat, 2007)
	The reasons provided by the AI shopping assistant for its recommendations are easy to understand.	
	The AI shopping assistant explains how product features correspond to my needs.	
	The explanations provided by the AI shopping assistant are sufficient to help me judge whether the recommendations are reasonable.	
Perceived Responsiveness (PR)	The AI shopping assistant responds promptly to my questions and requests.	(Q. Chen et al., 2022; J. Li et al., 2023)
	The AI shopping assistant’s responses closely address the questions I ask.	
	The AI shopping assistant understands my follow-up questions and responds accordingly.	
	The AI shopping assistant maintains a coherent conversation across multiple rounds of interaction.	
Perceived Usefulness (PU)	Using the AI shopping assistant improves the efficiency of my search for suitable products.	(Davis, 1989)
	Using the AI shopping assistant enables me to compare different products more effectively.	
	Using the AI shopping assistant helps me make better product choices.	
	Overall, the AI shopping assistant is useful for completing this shopping task.	
Algorithmic Trust (AT)	I believe this AI shopping assistant has the expertise needed to provide product recommendations.	(Jian et al., 2000; Wang & Benbasat, 2007)
	I believe the product suggestions provided by this AI shopping assistant are generally reliable.	
	I believe this AI shopping assistant provides recommendations in a consistent and reasonable manner.	
	I believe this AI shopping assistant does not intentionally withhold important information that could affect its recommendations.	
Privacy Concern (PC)	I am concerned that the AI shopping assistant collects more personal information than is necessary to complete this recommendation task.	(Malhotra et al., 2004; Smith et al., 1996)
	I am concerned that my conversations with the AI shopping assistant may be used for purposes beyond this shopping task.	
	I am concerned that I do not have sufficient control over the storage and use of my conversation data.	
	I am concerned that the AI shopping assistant may infer personal preferences or consumption-related information that I do not wish to disclose from our conversations.	
Purchase Intention (PI)	I would seriously consider purchasing the product ultimately recommended by the AI shopping assistant.	(Pavlou, 2003; Song & Shin, 2024)
	I am likely to purchase the product ultimately recommended by the AI shopping assistant.	
	When I have a corresponding shopping need, I would give priority to the product ultimately recommended by the AI shopping assistant.	
	Overall, I intend to purchase the product ultimately recommended by the AI shopping assistant.	

**Table 2:** Reliability and convergent validity of the measurement model.

Construct/Item	Estimate	Cronbach’s α	CR	AVE
IQ1	0.741	0.844	0.844	0.575
IQ2	0.743			
IQ3	0.786			
IQ4	0.762			
PP1	0.760	0.847	0.847	0.581
PP2	0.783			
PP3	0.761			
PP4	0.744			
PE1	0.747	0.858	0.858	0.602
PE2	0.776			
PE3	0.840			
PE4	0.737			
PR1	0.749	0.854	0.854	0.595
PR2	0.757			
PR3	0.770			
PR4	0.807			
PU1	0.776	0.850	0.851	0.588
PU2	0.777			
PU3	0.723			
PU4	0.789			
AT1	0.768	0.848	0.849	0.584
AT2	0.788			
AT3	0.690			
AT4	0.806			
PC1	0.770	0.849	0.848	0.584
PC2	0.764			
PC3	0.789			
PC4	0.731			
AIL4	0.769	0.867	0.867	0.567
AIL3	0.740			
AIL2	0.822			
AIL1	0.745			
AIL5	0.683			
PI1	0.738	0.845	0.845	0.577
PI2	0.794			
PI3	0.747			
PI4	0.758			

Note. Estimate = standardized factor loading; CR = composite reliability; AVE = average variance extracted.

**Table 3:** KMO and Bartlett’s Test.

KMO	0.943	
Bartlett’s sphericity	Spherical test	11,438.016
	df-value	666.000
	*p*-value	<0.001

Note. KMO = Kaiser–Meyer–Olkin measure of sampling adequacy.

**Table 4:** Discriminant validity: Fornell–Larcker criterion.

	PI	AIL	PC	AT	PU	PR	PE	PP	IQ
PI	0.760								
AIL	0.393	0.753							
PC	0.236	0.522	0.764						
AT	0.546	0.441	0.491	0.764					
PU	0.532	0.453	0.468	0.464	0.767				
PR	0.321	0.456	0.540	0.457	0.487	0.771			
PE	0.355	0.545	0.593	0.511	0.557	0.620	0.776		
PP	0.490	0.549	0.684	0.568	0.544	0.509	0.614	0.762	
IQ	0.480	0.443	0.566	0.525	0.510	0.440	0.524	0.620	0.758

Note. The diagonal elements represent the square root of the AVE for each construct, while the off-diagonal elements represent inter-construct correlations.

**Table 5:** Discriminant validity: HTMT ratios.

	PI	AIL	PC	AT	PU	PR	PE	PP	IQ
PI									
AIL	0.398								
PC	0.236	0.519							
AT	0.545	0.434	0.488						
PU	0.531	0.449	0.474	0.462					
PR	0.320	0.453	0.537	0.457	0.488				
PE	0.359	0.532	0.593	0.506	0.564	0.620			
PP	0.489	0.551	0.681	0.560	0.548	0.509	0.603		
IQ	0.478	0.445	0.563	0.525	0.513	0.433	0.519	0.621	

Note. The values represent heterotrait–monotrait (HTMT) ratios between constructs.

**Table 6:** Model fit indices for confirmatory factor analysis.

Category	Fit Index	Fit Criterion	Model Result	Model Fit Status
Absolute fit indices	CMIN/DF	<3	1.307	Yes
	GFI	>0.9	0.936	Yes
	AGFI	>0.9	0.924	Yes
	RMSEA	≤0.05	0.023	Yes
Incremental fit indices	NFI	>0.9	0.934	Yes
	TLI	>0.9	0.981	Yes
	IFI	>0.9	0.984	Yes
	CFI	>0.9	0.983	Yes
	RFI	>0.9	0.926	Yes
Parsimonious fit indices	PCFI	≥0.5	0.876	Yes
	PNFI	≥0.5	0.831	Yes
	PGFI	≥0.5	0.789	Yes

Note. CMIN/DF = chi-square divided by degrees of freedom; GFI = goodness-of-fit index; AGFI = adjusted goodness-of-fit index; RMSEA = root mean square error of approximation; NFI = normed fit index; TLI = Tucker–Lewis index; IFI = incremental fit index; CFI = comparative fit index; RFI = relative fit index; PCFI = parsimonious comparative fit index; PNFI = parsimonious normed fit index; PGFI = parsimonious goodness-of-fit index.

**Table 7:** Hypothesis test results.

Hypothesis/Path	Estimate	S.E.	C.R.	*p*	Testing the Hypothesis
H1a IQ → PU	0.189	0.06	3.214	0.001 **	Significant
H1b PP → PU	0.218	0.07	3.183	0.001 **	Significant
H1c PE → PU	0.228	0.067	3.516	<0.001 ***	Significant
H1d PR → PU	0.144	0.057	2.552	0.011 *	Significant
H2a IQ → AT	0.21	0.06	3.546	<0.001 ***	Significant
H2b PP → AT	0.303	0.07	4.347	<0.001 ***	Significant
H2c PE → AT	0.134	0.065	2.077	0.038 *	Significant
H2d PR → AT	0.118	0.057	2.093	0.036 *	Significant
H3 PP → PC	0.73	0.055	13.974	<0.001 ***	Significant
H4a PU → PI	0.322	0.055	5.638	<0.001 ***	Significant
H4b AT → PI	0.336	0.056	5.883	<0.001 ***	Significant
H4c PC → PI	−0.350	0.066	−5.002	<0.001 ***	Significant
H5a IQ → PI	0.169	0.06	2.797	0.005 **	Significant
H5b PP → PI	0.324	0.096	3.341	<0.001 ***	Significant
Residual direct path PE → PI	−0.096	0.065	−1.470	0.141	Not significant
Residual direct path PR → PI	−0.008	0.055	−0.137	0.891	Not significant

Note. Estimate = standardized path coefficient. S.E., C.R., and *p* values are based on the corresponding unstandardized regression estimates. S.E. = standard error; C.R. = critical ratio. *** indicates *p* < 0.001; ** indicates *p* < 0.01; * indicates *p* < 0.05.

**Table 8:** Total indirect, direct and total effects.

Mediation Path	Effect	Estimate	Lower	Upper	*p*
IQ—(PU/AT)—PI	Total Indirect Effect	0.131	0.075	0.204	<0.001
	Direct Effect	0.169	0.048	0.287	0.005
	Total Effect	0.300	0.175	0.419	<0.001
PP—(PU/AT/PC)—PI	Total Indirect Effect	−0.084	−0.205	0.041	0.195
	Direct Effect	0.324	0.154	0.498	<0.001
	Total Effect	0.241	0.092	0.395	0.002
PE—(PU/AT)—PI	Total Indirect Effect	0.118	0.052	0.196	0.001
	Direct Effect	−0.096	−0.232	0.030	0.144
	Total Effect	0.023	−0.114	0.157	0.734
PR—(PU/AT)—PI	Total Indirect Effect	0.086	0.029	0.153	0.004
	Direct Effect	−0.008	−0.119	0.097	0.882
	Total Effect	0.078	−0.037	0.192	0.173

Note. Effect estimates are standardized. Lower and Upper represent the lower and upper bounds of the 95% bias-corrected bootstrap confidence intervals based on 5000 bootstrap samples, respectively. The terms “direct effect,” “indirect effect,” and “total effect” are retained as conventional SEM terminology for model-based estimates and should not be interpreted as establishing causal effects or temporal mediation in the present cross-sectional design.

**Table 9:** Specific indirect effects.

Hypothesis/Path	Effect	Estimate	Lower	Upper	*p*	Testing the Hypothesis
H6a: IQ → PU → PI	Specific Indirect Effect	0.061	0.026	0.106	<0.001 ***	Supported
H7a: IQ → AT → PI		0.070	0.033	0.122	<0.001 ***	Supported
H6b: PP → PU → PI		0.070	0.027	0.131	0.001 **	Supported
H7b: PP → AT → PI		0.102	0.051	0.169	<0.001 ***	Supported
H8: PP → PC → PI		−0.255	−0.356	−0.165	<0.001 ***	Supported
H6c: PE → PU → PI		0.073	0.031	0.129	<0.001 ***	Supported
H7c: PE → AT → PI		0.045	0.004	0.095	0.033 *	Supported
H6d: PR → PU → PI		0.046	0.011	0.094	0.010 *	Supported
H7d: PR → AT → PI		0.040	0.002	0.087	0.039 *	Supported

Note. Effect estimates are standardized specific indirect effects. Lower and Upper represent the lower and upper bounds of the 95% bias-corrected bootstrap confidence intervals based on 5000 bootstrap samples, respectively. *** indicates *p* < 0.001; ** indicates *p* < 0.01; * indicates *p* < 0.05. The terms “direct effect,” “indirect effect,” and “total effect” are retained as conventional SEM terminology for model-based estimates and should not be interpreted as establishing causal effects or temporal mediation in the present cross-sectional design.

**Table 10:** Moderation analysis of AIL.

Hypothesis/Path	Effect Size (B)	SE	t	*p*	LLCI	ULCI
H9a PU × AIL → PI	0.0142	0.0299	0.4757	0.6345	−0.0444	0.0728
H9b AT × AIL → PI	0.1307	0.0302	4.3318	<0.001	0.0714	0.1900
H9c PC × AIL → PI	−0.1330	0.0269	−4.9410	<0.001	−0.1859	−0.0801

Note. B = unstandardized interaction coefficient; SE = standard error; LLCI and ULCI represent the lower and upper bounds of the 95% confidence interval, respectively. AIL = AI Literacy.

## Data Availability

The datasets used and analyzed during this study are available upon reasonable request to the corresponding author. The data are not publicly available due to privacy and confidentiality considerations related to the survey respondents.

## Abbreviations

The following abbreviations are used in this manuscript:

ICH	Intangible Cultural Heritage
SOR	Stimulus–Organism–Response
CB-SEM	Covariance-Based Structural Equation Modeling
IQ	Information Quality
PP	Perceived Personalization
PE	Perceived Explainability
PR	Perceived Responsiveness
PU	Perceived Usefulness
AT	Algorithmic Trust
PC	Privacy Concern
PI	Purchase Intention
AIL	AI Literacy

## Appendix A

**Table A1:** Demographic Characteristics of the Final Sample.

Characteristic	Category	n	%
Gender	Male	256	43.54%
	Female	332	56.46%
Age	18–25	98	16.67%
	26–35	209	35.54%
	36–45	193	32.82%
	46+	88	14.97%
Education	High school or below	69	11.73%
	Associate degree	96	16.33%
	Bachelor degree	320	54.42%
	Master degree or above	103	17.52%
Income (RMB)	Below 3000	56	9.52%
	3001–5000	118	20.07%
	5001–8000	212	36.05%
	8001–12,000	125	21.26%
	Above 12,000	77	13.10%
Shopping Frequency	Daily or almost daily	110	18.71%
	Several times a week	220	37.41%
	Several times a month	195	33.16%
	Rarely	63	10.71%
Total		588	100%

**Table A2:** Sequential Diagnostic Analysis of the Privacy Concern–Purchase Intention Coefficient.

Model	Sequential Specification	Standardized PC → PI Coefficient (β)	Pattern
Model 1	PC → PI only	0.236	Positive
Model 2	PC + PU → PI	0.030	Attenuated to near zero
Model 3	PC + PU + AT → PI	−0.107	Sign reversal emerges
Model 4	Model 3 + PP → PC + PP → PI	−0.308	Negative coefficient strengthens
Model 5	Original full SEM	−0.350 ***	Final conditional coefficient

Note. Models 1–4 are supplementary sequential latent-variable SEM specifications estimated using the original data. Model 1 estimates the PC–PI association; Model 2 additionally includes PU as a predictor of PI; Model 3 additionally includes AT; and Model 4 incorporates PP and its specified associations with PU, AT, and PC. Model 5 reports the PC → PI coefficient from the original full structural model. Standardized coefficients are reported. *** *p* < 0.001.

**Table A3:** Collinearity Diagnostics for Predictors of Purchase Intention.

Predictor	Tolerance	VIF
Information Quality (IQ)	0.621	1.610
Perceived Personalization (PP)	0.514	1.944
Perceived Explainability (PE)	0.555	1.803
Perceived Responsiveness (PR)	0.651	1.536
Perceived Usefulness (PU)	0.656	1.524
Algorithmic Trust (AT)	0.672	1.488
Privacy Concern (PC)	0.631	1.585

Note. Collinearity diagnostics are based on a multiple regression using composite scores, with purchase intention as the dependent variable and all seven predictors entered simultaneously.

**Table A4:** Comparison of Alternative Structural Models.

Model	χ^2^	df	CFI	TLI	RMSEA	AIC	Nested Comparison
Model 1: Parallel	631.874	442	0.980	0.978	0.027	803.874	—
Model 2: +PU → AT	628.954	441	0.980	0.978	0.027	802.954	vs. M1: Δχ^2^(1) = 2.920, *p* = 0.087
Model 3: Sequential	735.956	445	0.969	0.966	0.033	901.956	vs. M2: Δχ^2^(4) = 107.002, *p* < 0.001

Note. Model 1 is the focal parallel specification. Model 2 adds the PU → AT path while retaining the four direct cue → AT paths. Model 3 retains PU → AT but constrains the four direct cue → AT paths to zero. Model 1 and Model 2 are nested, as are Model 2 and Model 3. Model 1 and Model 3 are not treated as a standard nested χ^2^-difference comparison.

**Table A5:** Equality-Constraint Tests for Selected Direct Structural Paths.

Comparison	Unconstrained Coefficients (β)	Constrained Model χ^2^	df	Δχ^2^	Δdf	*p*
IQ → PI vs. PE → PI	0.169 vs. −0.096	640.438	443	8.564	1	0.003
PP → PI vs. PR → PI	0.324 vs. −0.008	641.972	443	10.098	1	0.001

Note. The unconstrained baseline model had χ^2^ = 631.874 with df = 442. Equality constraints were imposed on the corresponding unstandardized structural regression coefficients, whereas the standardized coefficients (β) from the unconstrained model are reported in the table for interpretation. Each constrained model was compared with the unconstrained baseline model using a chi-square difference test. A significant Δχ^2^ indicates that constraining the corresponding pair of structural coefficients to equality significantly worsened model fit.

**Table A6:** Supplementary Equivalence Test for the PU × AI Literacy Interaction.

Interaction	B	SE	Lower Bound	Upper Bound	Lower-Bound Test	Upper-Bound Test	Interpretation
PU × AIL	0.0142	0.0299	−0.10	0.10	t = 3.82, *p* < 0.001	t = −2.87, *p* < 0.01	Within specified equivalence bounds

Note. B = unstandardized interaction coefficient; SE = standard error. The equivalence bounds of −0.10 and 0.10 represent the specified study-specific practical-equivalence criterion for this supplementary analysis and are not intended as a universally accepted small-effect threshold.
